# Bioprocessing of Squid Pens Waste into Chitosanase by *Paenibacillus* sp. TKU047 and Its Application in Low-Molecular Weight Chitosan Oligosaccharides Production

**DOI:** 10.3390/polym12051163

**Published:** 2020-05-19

**Authors:** Chien Thang Doan, Thi Ngoc Tran, Van Bon Nguyen, Trung Dung Tran, Anh Dzung Nguyen, San-Lang Wang

**Affiliations:** 1 Department of Chemistry, Tamkang University, New Taipei City 25137, Taiwan; doanthng@gmail.com (C.T.D.); ttngoc@ttn.edu.vn (T.N.T.); 2 Department of Science and Technology, Tay Nguyen University, Buon Ma Thuot 630000, Vietnam; ttddhtn@gmail.com; 3 Institute of Research and Development, Duy Tan University, Da Nang 550000, Vietnam; nguyenvanbon@duytan.edu.vn; 4 Institute of Biotechnology and Environment, Tay Nguyen University, Buon Ma Thuot 630000, Vietnam; nadzungtaynguyenuni@yahoo.com.vn; 5 Life Science Development Center, Tamkang University, New Taipei City 25137, Taiwan

**Keywords:** chitosan, chitosanase, chitosan oligosaccharides, *Paenibacillus*, free radical scavenging activity

## Abstract

Chitosan oligosaccharide (COS) has become of great interest in recent years because of its worthy biological activities. This study aims to produce COS using the enzymatic method, and investigates *Paenibacillus* sp. TKU047, a chitinolytic-producing strain, in terms of its chitosanase productivity on several chitinous material-containing mediums from fishery process wastes. The highest amount of chitosanase was produced on the medium using 2% (*w*/*v*) squid pens powder (0.60 U/mL) as the single carbon and nitrogen (C/N) source. The molecular mass of TKU047 chitosanase, which could be the smallest one among chitinases/chitosanases from the *Paenibacillus* genus, was approximately 23 kDa according to the sodium dodecyl sulfate-polyacrylamide gel electrophoresis (SDS-PAGE) method. TKU047 chitosanase possessed the highest activity at 60 °C, pH 7, and toward chitosan solution with a higher degree of deacetylation (DDA) value. Additionally, the hydrolysis products of 98% DDA chitosan catalyzed by TKU047 chitosanase showed the degree of polymerization (DP) ranging from 2 to 9, suggesting that it was an endo-type activity chitosanase. The free radical scavenging activity of the obtained chitosan oligosaccharide (COS) was determined. The result showed that COS produced with *Paenibacillus* sp. TKU047 chitosanase expressed a higher 2,2-diphenyl-1-picrylhydrazyl (DPPH) radical scavenging activity than that from the commercial COSs with maximum activity and IC_50_ values of 81.20% and 1.02 mg/mL; 18.63% and 15.37 mg/mL; and 15.96% and 15.16 mg/mL, respectively. As such, *Paenibacillus* sp. TKU047 may have potential use in converting squid pens waste to produce chitosanase as an enzyme for bio-activity COS preparation.

## 1. Introduction

Chitosan is a linear polymer that is produced from chitin via the deacetylation process [1]. Chitosan comprises some excellent properties including non-toxicity, adsorption, biocompatibility, and biodegradability, thus, it can potentially be used in many fields such as medicine, wastewater treatment, agriculture, and functional food [2,3,4,5,6,7]. One of the most challenging applications of chitosan is its poor solubility in a neutral aqueous solution. Therefore, chitosan oligosaccharide (COS), which shows a higher solubility in neutral water than chitosan, is receiving great attention. COS exhibits numerous properties, such as antifungal, antimicrobial, antitumor, antioxidant, immuno-enhancing, anti-inflammatory, and antidiabetic activities [8,9,10,11,12,13,14,15,16,17]. The three major processes to convert chitosan into COS include physical, chemical, and enzymatic methods. Compared to physical and chemical methods, the enzymatic method seems to be more advantageous because it enables controlling the molecular mass of the COS product and the reaction condition is gentler [2]. However, the biggest obstacle to adopting this method is the expensive cost of the enzyme. To reduce the cost of the enzyme and that of COS produced by the enzymatic method, some solutions include reducing the enzyme purification steps [18], using a commercial enzyme complex to hydrolyze chitosan [19], immobilizing the enzyme [20], using ultrafiltration membrane enzymatic system [21], and utilizing low-cost materials to produce chitinolytic enzymes via microbial fermentation [22]. 

Chitosanase is a group of hydrolytic enzymes that catalyze the breakdown of 1,4 glucoside bonds of chitosan. This enzyme can be split into endo-type activity (endo-chitosanase) and exo-type activity (exo-1,4-β-D-glucosaminidase) based on its cleavage site on the substrate [2]. Endo-chitosanase degrades chitosan at a random position and releases COS, thus it is the major enzyme responsible for COS production [2]. Until now, numerous bacteria have been explored for their chitosanase producing ability, such as *Bacillus* [10,15,18,23,24,25,26,27,28,29], *Paenibacillus* [8,13,30,31,32,33,34,35,36,37,38,39,40,41], *Acinetobacter* [42,43], *Streptomyces* [14,44], *Serratia* [45], and *Pseudomonas* [46]. For the production of chitosanase via bacterial fermentation, chitin is the common carbon and nitrogen (C/N) supplement. To produce chitin from chitinous materials by a chemical process, a series of steps named deproteinization (using an alkali), or demineralization (using an acid), must be performed [2,3,4]. However, some drawbacks occur during or after the chitin and chitosan preparations, such as the reduction of chitin quality and the emissions of polluting wastewater [2]. Fishery processing by-products—squid pens, crab shells, and shrimp shells, for example—are the main sources for chitin and chitosan [4,20]. In addition to the chitin component, they also contain a significant amount of protein and mineral salts [4,47]. Thus, these chitinous materials can be directly used as the source of nutrition for microbial fermentation to produce chitinolytic enzymes [8,10,13,14,15] as well as other valuable products [48,49,50,51,52,53,54,55,56,57].

*Paenibacillus* was separated from the *Bacillus* genus in 1993 with around 200 species [58]. Many strains of this genus have revealed various biological activities, with potential applications in medical, agricultural, and industrial sectors [8,13,30,31,32,33,34,35,36,37,38,39,40,41,54,57,58]. Recently, marine chitinous wastes including shrimp heads, crab shells, and squid pens were extensively used for the production of chitinolytic enzymes, proteases, antidiabetic drugs, and exopolysaccharides by *Paenibacillus* species [4]. However, there is not much research on the conversion of marine chitinous wastes, especially squid pens, to produce chitosanase via this genus. *P. pasadenensis* CS0611 and *P. ehimensis* MA2012 can produce chitinase on the medium containing crab shells along with some other C/N sources such as peptone, gelatin, sucrose, and yeast extract [39,59]. *P. mucilaginosus* TKU032 exhibited the highest chitosanase productivity on the medium containing only shrimp heads as the C/N source, while *P. macerans* TKU029 and *Paenibacillus* sp. TKU042 exhibited the best productivity using squid pens as the C/N source [8,13,31]. Thus, utilizing marine chitinous wastes is of interest to produce a chitosanase from *Paenibacillus* sp. TKU047, which was isolated using medium containing squid pen as the single C/N source [54].

In the current study, the chitosanase productivity of *Paenibacillus* sp. TKU047 using several chitinous materials, such as shrimp heads, shrimp shells, demineralized shrimp shells, demineralized crab shells, squid pens, and commercial chitin was evaluated to find the appropriate C/N source for the enzyme production process. Then, *Paenibacillus* sp. TKU047 chitosanase was purified and its property was characterized. Furthermore, COS, produced by the hydrolysis of chitosan with *Paenibacillus* sp. TKU047 chitosanase was extracted and analyzed by MALDI-TOF mass spectrometry. The antioxidant activity of the obtained COS was also evaluated and compared with that of commercial COSs.

## 2. Materials and Methods

### 2.1. Materials

*Paenibacillus* sp. TKU047 strain was earlier isolated and obtained from previous research [54]. Shrimp heads were obtained from Fwu-Sow Industry (Taichun, Taiwan). Crab shells, shrimp shells, and squid pens were bought from Shin-Ma Frozen Food Co. (I-Lan, Taiwan). Demineralization of crab shells and shrimp shells was carried out using an acid method. Briefly, shrimp head and crab shell were treated with HCl solution (2 N) for two days and then rinsed with water and dried in an oven at 65 °C. Water-soluble chitosan (a hydrochloride salt, average molecular weight or MW ≈ 12.5 kDa and degree of deacetylation or DDA ≈ 76%), chitosan with 50%–70% of DDA (average MW ≈ 80 kDa), 70%–90% of DDA (average MW ≈ 52 kDa), and 98% DDA chitosan (average MW ≈ 40 kDa) were provided by the Microorganisms and Biochemistry Laboratory, Department of Chemistry, Tamkang University, New Taipei, Taiwan. Chitin from shrimp shells, ≥75% DDA chitosan, 2,2-diphenyl-1-picrylhydrazyl (DPPH), and 3,5-dinitrosalicylic acid (DNS) reagents were all bought from Sigma Co. (St. Louis, MO, USA). Food-grade chitosan oligosaccharide (CCOS_1) and chitosan oligosaccharide X13 (CCOS_2) were bought from Charming & Beauty Co. (Taipei, Taiwan). Other chemicals were of the highest purity obtainable.

### 2.2. Chitosanase Assay

The chitosanase assay was performed as described in the previous study [8]. The chitosan hydrolysis reaction was carried out in a glass tube with an equal volume of substrate (0.1 mL of 1% *w*/*v* chitosan) and the sample (0.1 mL) for 30 min at 37 °C of incubation temperature. Then, the mixture was added to 1.5 mL of the DNS reagent and heated at 100 °C for 10 min and the developed color was measured using an ELISA plate reader to determine the chitosanase of the sample. One unit of chitosanase activity was the amount of enzyme needed to produce 1 µmol of reducing sugar in one minute.

### 2.3. Screening of Suitable C/N Source for Chitosanase Production

One percent of shrimp heads powder (SHP), shrimp shells powder (SSP), demineralized shrimp shells powder (deSSP), demineralized crab shells powder (deCSP), squid pens powder (SPP), and chitin powder were added to the medium containing 0.05% MgSO4.7H_2_O and 0.1% K_2_HPO_4_ to provide the C/N source for the fermentation of *Paenibacillus* sp. TKU047. The medium was prepared in a 250 mL flask and sterilized in an autoclave at 121 °C for 30 min before being used. One percent of seed solution was added to every medium flask to start the fermentation at 37 °C and 150 rpm shaking speed for 5 days. After every 24 h, 1 mL of culture medium was withdrawn to examine the chitosanase activity. To investigate the optimal concentration of C/N source for the chitosanase production, the amount of SPP was adjusted in the range of 0.5% to 2.5% (*w*/*v*), while other components (MgSO_4_.7H_2_O and K_2_HPO_4_) were similar to their initial concentrations as mentioned above.

### 2.4. Isolation of Paenibacillus sp. TKU047 Chitosanase

To isolate the chitosanase, 200 mL of the medium from the 4-day culture of *Paenibacillus* sp. TKU047 were collected and centrifuged at 6000 rpm for 30 min to remove the residual solids. The culture supernatant was gently mixed with 120 g of ammonium sulfate and kept at 4 °C for one day. The crude enzyme was separated from the mixture by centrifuging at 9000 rpm for 30 min and the pellet was then dissolved in a small amount of 50 mM sodium phosphate buffer (pH 5.8). The residual ammonium sulfate in the crude enzyme was removed by dialysis against 50 mM sodium phosphate buffer (pH 5.8) for 24 h using a cellulose dialysis membrane (Cellu Sep T2, molecular-weight cutoff of 6000–8000 Da, Membrane Filtration Products, Inc., Seguin, TX, USA). Next, the crude enzyme was injected into a column containing strong cation exchange resin (Macro-Prep High S) as the stationary phase equilibrated with 50 mM sodium phosphate buffer (pH 5.8). The elution was performed by using a gradient of NaCl (from 0 to 0.5 M), prepared in a similar buffer. For each tube, the chitosanase assay was performed to find the fraction which expressed the chitosanase activity. The fractions which showed enzyme activity were then concentrated by lyophilization. The obtained enzyme powder was dissolved in a small amount of distilled water and purification was continued by gel filtration using a high-performance liquid chromatography (HPLC) system with KW802.5 column (Showa Denko K. K, Tokyo, Japan). The molecular mass of the purified chitosanase was determined using SDS-PAGE. The protein bands were stained by Protein Assay Dye Reagent from BioRad (Hercules, CA, USA). A zymogram of chitosanase was performed on acrylamide gel containing 0.01% of chitosan. After electrophoresing, the gel was washed with 2% Triton X-100 and then with sodium phosphate buffer. The hydrolysis reaction of chitosanase was performed by keeping the gel in a similar buffer at 37 °C for 12 h. The bands indicating chitosanase activity were visualized by 0.1% (*w*/*v*) congo red solution.

### 2.5. Effects of Temperature and pH

The optimal temperature of *Paenibacillus* sp. TKU047 chitosanase activity was determined by incubating 0.2 mL of the mixture of the purified chitosanase and the substrate at different temperatures (30 °C–90 °C) for 30 min. Later on, 1.5 mL of DNS reagent were added to the mixture and heated at 100 °C for 10 min to estimate the amount of reducing sugar. The thermal stability of *Paenibacillus* sp. TKU047 chitosanase was determined by incubating the enzyme solution at different temperatures for 60 min and then investigating the residual activity of tested enzyme solutions using the chitosanase assay (as described above).

The optimal pH for *Paenibacillus* sp. TKU047 chitosanase activity was determined by adding the enzyme into substrate solutions at different pH (3–11). The pH stability of the enzyme of *Paenibacillus* sp. TKU047 chitosanase was investigated based on its residual activity after pre-treating the enzyme at different pH points in 60 min using a buffer system, including pH 3 (glycine HCl); 4 and 5 (sodium acetate); 6, 7, and 8 (sodium phosphate); and 9, 10, and 11 (sodium bicarbonate-carbonate). The residual activity was performed at pH 7 and 37 °C in 30 min.

### 2.6. Effect of Divalent Metal Ions, Surfactants, and EDTA

Divalent metal ions solutions were prepared at 5 mM of the concentration, and surfactants (Triton X-100 and SDS) and EDTA were at 10% concentration. Initially, an equal volume of each of those chemicals and *Paenibacillus* sp. TKU047 chitosanase were mixed in a glass tube for 30 min and then the residual activities of TKU047 chitosanase were then tested by chitosanase assay (as described above).

### 2.7. Substrate Specificity and Hydrolysis Products

Chitin, cellulose, water-soluble chitosan, 50%–70% DDA chitosan, 70%–90% DDA chitosan, ≥75% DDA chitosan, and 98% DDA chitosan were used individually to test the substrate specificity of *Paenibacillus* sp. TKU047 chitosanase activity using the testing conditions as described above. The chitosanase activity in ≥75% DDA chitosan solution was used as the control. The hydrolysis solutions of 98% DDA chitosan with TKU047 chitosanase at 0 min, 30 min, 60 min, 120 min, and 180 min were analyzed by thin-layer chromatography (TLC). Here, the mobile phase was a mixture of propanol/ammonia solution/water (70/10/20, *v*/*v*/*v*). The hydrolysis products on the TLC plate were visualized by spraying the plate with 10% H_2_SO_4_ in ethanol and heating it at 180 °C.

### 2.8. Preparation of COS

The crude enzyme from the ammonium sulfate precipitation step was used to prepare COS. The crude enzyme was added to 98% DDA chitosan solution (1%, *w*/*v*) to start the hydrolysis reaction. The mixture was then incubated at 37 °C for 24 h. Then, the pH of the mixture was adjusted to neutral (pH 7) to precipitate the residual amount of chitosan and the mixture was centrifuged at 6000 rpm for 30 min. The supernatant was collected and used to extract COS via selective precipitation method using methanol and acetone [14]. In short, methanol was added to the supernatant in a ratio of 9/1 (*v*/*v*) to precipitate the high molecular weight particles, which were removed by centrifuging at 9000 rpm for 30 min. The supernatant was then concentrated by a rotary evaporator to reach 1/10 of its initial volume. Eventually, the COS was collected by adding acetone into the supernatant (9/1, *v*/*v*) and centrifuging (9000 rpm for 30 min) the mixture to obtain the precipitate.

The obtained COS was then analyzed by MALDI-TOF mass spectrometry (Bruker Daltonics, Bremen, Germany) with a UV laser (337 nm) [14]. A solution of 15 mg/mL of 2,5–dihydroxybenzoic acid in 30% aqueous ethanol was used as the matrix substance. For each spectrum, the data of 30–50 laser shots were acquired and analyzed.

### 2.9. Antioxidant Activity Assay

To test for antioxidant activity, solutions of COS, CCOS_1, and CCOS_2 were prepared in a range of concentration from 0.16 mg/mL to 20 mg/mL. Antioxidant activity was assayed as per the DPPH radical scavenging activity method, described in a previous study [8].

### 2.10. Statistical Analysis

The data are shown as mean ± standard deviation of three replications. Statistical analysis was performed by one-way ANOVA analysis using Microsoft Office Excel.

## 3. Results and Discussion

### 3.1. Screening of Suitable C/N Source for Chitosanase Production by Paenibacillus sp. TKU047

**Figure 1:**
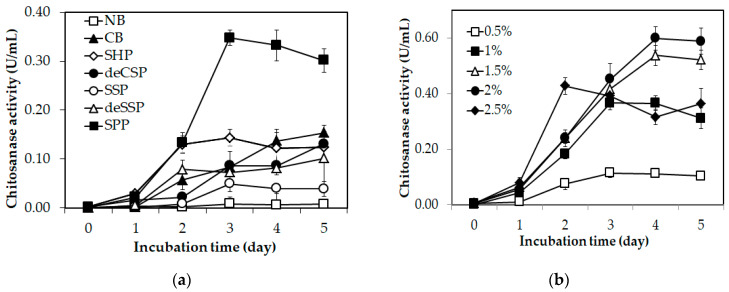
Screening of suitable C/N source for chitosanase production by *Paenibacillus* sp. TKU047: (**a**) different chitinous sources and (**b**) different concentrations of SPP. NB: nutrient broth; CP: chitin powder; SHP: shrimp heads powder; deCSP: demineralized crab shells powder; SSP: shrimp shells powder; deSSP: demineralized shrimp shells powder; and SPP: squid pens powder. All data points were the mean and standard deviation.

Six types of chitin sources, including five types from fishery wastes (shrimp heads powder (SHP), shrimp shells powder (SSP), demineralized shrimp shells powder (deSSP), demineralized crab shells powder (deCSP), and squid pens powder (SPP)) and a commercial chitin powder (CP) from Sigma Co. (St. Louis, MO, USA) were used to test the chitosanase production ability of *Paenibacillus* sp. TKU047. One gram of each chitin source was added to 100 mL of liquid medium containing MgSO_4_ (0.05 g) and KH_2_PO_4_ (0.1 g) to provide the carbon and nitrogen sources for the chitosanase synthesis by the bacterial strain. Nutrient broth (NB), a commercial medium from Himedia Co. (Mumbai, India), was used as a control medium for the experiment. As shown in Figure 1a, *Paenibacillus* sp. TKU047 exhibited chitosanase activity on the culture media containing chitinous sources and non-activity on NB (a non-chitinous medium) indicating that chitin was a key factor for chitosanase production by *Paenibacillus* sp. TKU047. In some bacterial strains, chitin/chitosan is required as a chitinase/chitosanase inducer [2,38]. Thus, the presence of chitin/chitosan in the culture medium could promote the chitinase/chitosanase synthesis process by the bacterium. Among all chitinous sources, SPP was found to be the most suitable supplement and *Paenibacillus* sp. TKU047 could express the highest chitosanase activity (0.35 ± 0.02 U/mL on the 3rd day of the fermentation period) in this medium compared to the activities on CP (0.15 ± 0.02 U/mL on the 5th day), SHP (0.14 ± 0.02 U/mL on the 3rd day), deCSP (0.13 ± 0.02 U/mL on the 5th day), SSP (0.05 ± 0.02 U/mL on the 3rd day), and deSSP (0.10 ± 0.06 on the 5th day). A similar phenomenon was observed in other reports, which showed that SPP was the most suitable chitinous source for chitosanase production via *Paenibacillus* strains [13,31]. Chitin has been a common supplement for chitinase/chitosanase production via microbial strains [4,14,36,37,38]. However, the cost of chitin may be a potential limitation for its use in chitinase/chitosanase production. Therefore, chitinous materials from fishery wastes such as squid pens, shrimp heads, and shrimp shells have been considered as an effective alternative [4,60]. Thus, by expressing a better result, SPP could be selected as the sole C/N source for chitosanase production by *Paenibacillus* sp. TKU047.

After confirming SPP as an important component in chitosanase production, the optimum concentration of SPP was also investigated. A higher SPP concentration, in the range 0.5%–2.0% (*w*/*v*), gave better chitosanase production results (Figure 1b). Particularly, the highest chitosanase activity was found on the 4th day of using 2% SPP (0.60 ± 0.04 U/mL) compared to 0.5% SPP (0.11 ± 0.02 U/mL on the 3rd day), 1% SPP (0.36 ± 0.01 U/mL on the 3rd day), and 1.5% SPP (0.54 ± 0.04 U/mL on the 4th day). Furthermore, when the SPP concentration increased to 2.5%, the chitosanase productivity of *Paenibacillus* sp. TKU047 decreased and the highest activity (0.43 ± 0.03 U/mL) was observed on the 2nd day of the fermentation period. Chitinases/chitosanases are inducible enzymes, indicating that the enzyme synthesis is strongly affected by medium composition [32]. Hence, according to the above-mentioned results, greater than 2% SPP concentrations may not suitable for the synthesis of chitosanase by *Paenibacillus* sp. TKU047. Therefore, 2% (*w*/*v*) of SPP was selected as the optimum concentration for producing chitosanase from *Paenibacillus* sp. TKU047.

### 3.2. Isolation of Paenibacillus sp. TKU047 Chitosanase

Isolation of the purified chitosanase was an essential step to further investigate its properties. The supernatant from the 4-day culture of *Paenibacillus* sp. TKU047 was collected and the crude enzyme was concentrated using the ammonium sulfate precipitation method. This was then purified using fast protein liquid chromatography (FPLC, using Macro-prep High S column) and high-performance liquid chromatography (HPLC, using KW802.5 column) methods. The summary of the purification procedure is presented in Table 1. Only one chitosanase fraction was observed during the purification process. The final purified chitosanase had a recovery yield of 4.87% and 80.28-folds of the specific activity.

**Figure 2:**
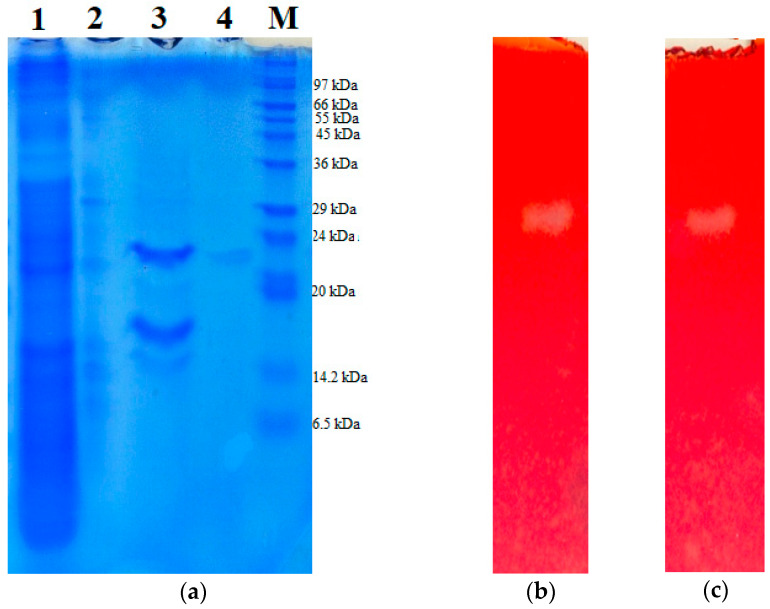
SDS-PAGE and zymogram profiles of *Paenibacillus* sp. TKU047 chitosanase. (**a**) SDS-PAGE; (**b**) Zymogram profile of culture supernatant; (**c**) Zymogram profile of purified *Paenibacillus* sp. TKU047 chitosanase. 1: Culture supernatant; 2: crude enzyme after (NH_4_)_2_SO_4_ precipitation; 3: chitosanase fraction after running fast protein liquid chromatography (FPLC) with Macro-prep High S column; 4: purified chitosanase after running high-performance liquid chromatography (HPLC) with KW802.5 column; and M: protein markers.

The molecular weight of the purified chitosanase was determined using SDS-PAGE. As shown in Figure 2a, the molecular weight of *Paenibacillus* sp. TKU047 chitosanase was nearly 23 kDa, indicating that it could be the smallest among the other chitinases/chitosanases from *Paenibacillus* species, with MW in the range of 30–70 kDa (Table 2). The enzyme activity was checked on a polyacrylamide gel containing 0.1% (*w*/*v*) chitosan. On visualizing with congo red solution, one chitinolytic activity band could be observed in the lane with the culture supernatant sample (Figure 2b), suggesting that *Paenibacillus* sp. TKU047 secreted only one chitosanase into the SPP medium. While most of the *Paenibacillus* strains produced one chitinase/chitosanase into the medium, there are several strains that produce more than one type of chitinase/chitosanase, such as *P. illinoisensis* KJA-424, *P. ehimensis* MA2012, *P. chitinolyticus* NP-306, and *Paenibacillus* sp. str. FPU-7 [37,59,61,62]. Here, on examining the band, one chitinolytic activity band in the lane of purified chitosanase located exactly in the same position as that in the lane of culture supernatant (Figure 2c), thus confirming that *Paenibacillus* sp. TKU047 chitosanase was successfully isolated.

### 3.3. Effects of Temperature and pH

**Figure 3:**
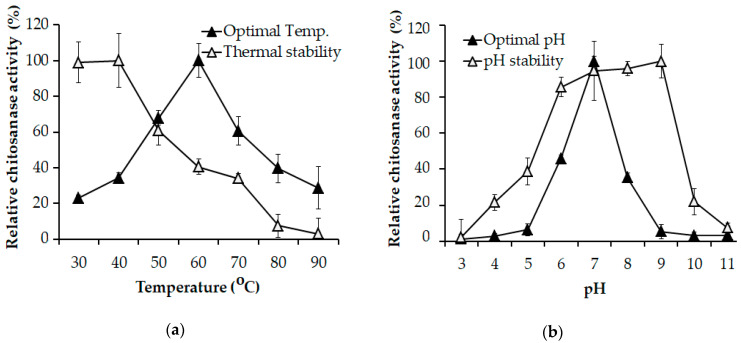
Effects of (**a**) temperature and (**b**) pH on the activity and stability of *Paenibacillus* sp. TKU047 chitosanase. All data points were the mean and standard deviation.

Thermal stability of *Paenibacillus* sp. TKU047 chitosanase was tested by treating the enzyme solution at different temperatures for 60 min. As shown in Figure 3a, *Paenibacillus* sp. TKU047 chitosanase retained the initial activity until 40 °C, and then significantly lost its activity at the higher temperatures. In this study, the optimum temperature of *Paenibacillus* sp. TKU047 chitosanase was observed to be 60 °C. Likewise, thermal stability and optimum temperature of chitinase/chitosanase from *Paenibacillus* strains have been reported at various temperatures from 37 °C to 80 °C [8,13,36,37,38,39,63,64,65].

The pH activity profile of *Paenibacillus* sp. TKU047 chitosanase is shown in Figure 3b. The optimum enzyme activity was observed at pH 7. In earlier studies, the optimum pH of chitosanase or chitinase from *Paenibacillus* strains such as *P. illinoisensis* KJA-424 [37], *Paenibacillus* sp. 1794 [30], *P. thermoaerophilus* TC22-2b [38], *Paenibacillus* sp. D1 [34], *P. mucilaginosus* TKU032 [8], and *P. pasadenensis* CS0611 [39] was commonly reported at acidic values. Nevertheless, there are some chitosanase/chitinase from *Paenibacillus* strains that show the optimum pH for an activity similar to that of *Paenibacillus* sp. TKU047 chitosanase (pH 7) such as *P. macerans* TKU029, *P. dendritiformis* and *P. elgii* HOA73 [13,33,63]. In this study, the pH stability of *Paenibacillus* sp. TKU047 chitosanase was found in a broad range from pH 6 to 9.

### 3.4. Effects of Divalent Metal Ions, EDTA, and Surfactants

**Figure 4:**
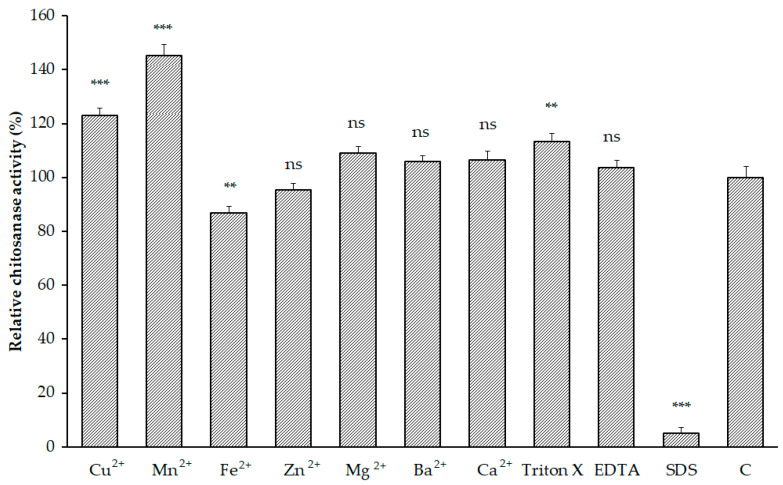
Effects of some chemicals on the activity of *Paenibacillus* sp. TKU047 chitosanase. All data points were the mean and standard deviation. Note: ns, **, *** were not significantly different, or significantly different at *p* < 0.01, or *p* < 0.001 (respectively). The activity of the enzyme incubated with the buffer was used as the control group.

The activity of *Paenibacillus* sp. TKU047 chitosanase incubated with some divalent metal ions was examined and the results are shown in Figure 4. Zn^2+^, Mg^2+^, Ba^2+^, and Ca^2+^ did not have a clear effect on enzyme activity. Fe^2+^ showed a slight inhibition effect by retaining 86.79% ± 2.43% of the initial enzyme activity, while two ions, Cu^2+^ and Mn^2+^, could enhance the activity of *Paenibacillus* sp. TKU047 chitosanase (122.90% ± 2.84% and 145.16% ± 4.31%, respectively). The effect of surfactants (Triton X-100 and SDS) on the enzyme activity was also carried on. Triton X-100 could enhance the activity of *Paenibacillus* sp. TKU047 by 113.16% ± 3.26%, whereas SDS completely inhibited the enzyme activity (reduced to 5.16% ± 1.97%). The enhancement effect of Triton X-100 may be related to the to the capacity of surface-active reagents to increase the frequency of contact between the substrate and the enzyme active site [67]. The negative effect of SDS on the enzyme activity may be attributed to its capacity to alter the enzyme secondary structure [68]. EDTA showed an insignificant effect on the activity of *Paenibacillus* sp. TKU047 chitosanase.

### 3.5. Substrate Specificity and Hydrolysis Products

**Figure 5:**
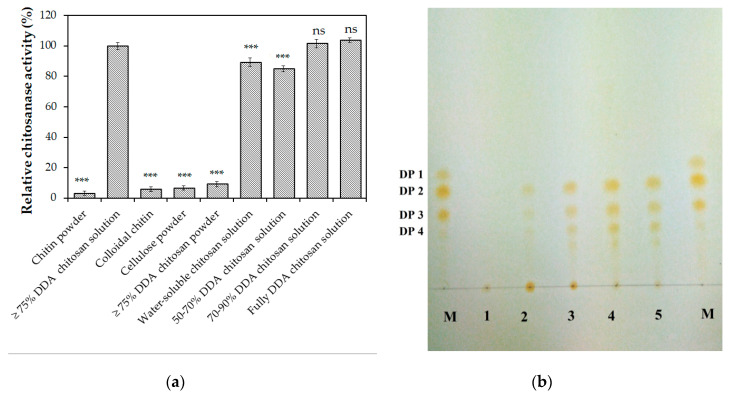
Substrates specificity of *Paenibacillus* sp. TKU047 chitosanase (**a**) and thin-layer chromatography (TLC) profile of hydrolysis products of 98% degree of deacetylation (DDA) chitosan by the purified enzyme (**b**). All data points were the mean and standard deviation. Note: ns, *** were not significantly different, or significantly different at *p* < 0.001 (respectively). The activity of the enzyme on ≥75% DDA chitosan solution was used as the control group. M: (GlcN)_1-4_; 1-6: the hydrolysis solutions were taken at 0 min, 30 min, 60 min, 120 min, and 180 min, respectively.

The activity of *Paenibacillus* sp. TKU047 chitosanase on different substrates was mentioned in Figure 5a. Among the tested substrates, TKU047 chitosanase exhibited a higher activity on high DDA chitosan solutions (100% ± 2.15% on >75% DDA chitosan, 101.52% ± 2.94% on 70%–90% DDA chitosan, and 103.66% ± 1.56% on 98% DDA chitosan) than on 50%–70% DDA chitosan solution, water-soluble chitosan (76% DDA) and colloidal chitin with activities 84.93% ± 1.86%, 89.27% ± 2.86% and 5.81% ± 1.61% (respectively) of the activity on >75% DDA chitosan, indicating the specificity of TKU047 chitosanase to the GlcN-GlcN bond. This phenomenon was observed earlier in a chitosanase from *P. dendritiformis* [63]. *Paenibacillus* sp. TKU047 chitosanase also showed insignificant chitinase and cellulase activities with less than 10% of its activity on chitin powder (3.17% ± 1.44%), colloidal chitin (5.81% ± 1.61%), and cellulose powder (6.65% ± 1.44%). The physical form of the substrate also affected the activity of TKU047 chitosanase. With the same source of chitosan (>75% DDA chitosan), the enzyme showed higher activity in the solution form (100% ± 2.15%) than that in the powder form (9.21% ± 1.86%).

The hydrolysis products by *Paenibacillus* sp. TKU047 chitosanase were determined using 98% DDA chitosan as the substrate. As shown in Figure 5b, the enzyme split chitosan into a mixture of GlcN oligomers with (GlcN)_2_, (GlcN)_3_, and (GlcN)_4_ as the primary products. Some trace spots, located under the spot for (GlcN)_4_ could also be observed, suggesting the presence of GlcN oligomers with a degree of polymerization (DP) higher than 4. The absence of GlcN product, as well as the presence of GlcN oligomers with a DP ≥ 2 as the major products in the initial period of the reaction time, indicated endo-type activity of the enzyme TKU047 chitosanase. This is concurrent with earlier reports wherein some chitinases/chitosanases from the *Paenibacillus* genus were confirmed as the endo-type enzymes [8,13,66].

### 3.6. Preparation of COS

**Figure 6:**
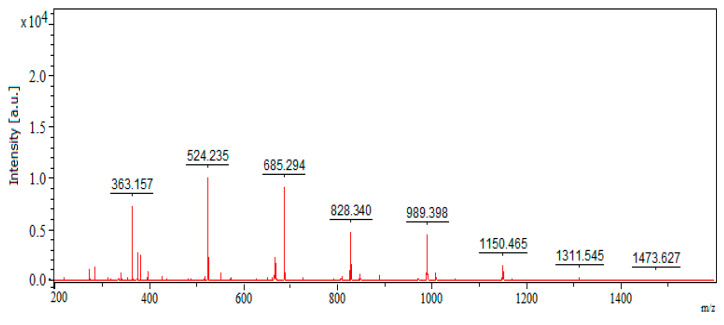
MALDI-TOF mass spectrometry profile of the chitosan oligosaccharide (COS) prepared from the chitosan hydrolysis process catalyzed by *Paenibacillus* sp. TKU047 chitosanase.

Chitosanase can be divided into endo-type activity and exo-type activity; endo-chitosanase is the major group responsible for chitosan oligosaccharide production [2]. After observing that *Paenibacillus* sp. TKU047 chitosanase is seemingly an endo-type activity enzyme, this enzyme could be considered a suitable tool to prepare COS via enzymatic method. To reduce the COS production cost, *Paenibacillus* sp. TKU047 crude enzyme from ammonium sulfate precipitation step was used to degrade chitosan. COS in the hydrolysis liquid of chitosan was isolated by selective precipitation method using methanol and acetone [14]. The obtained COS appeared as a yellowish powder with a yield of 68.44%. In previous studies, the COS production yield by the enzymatic method varied—for example, *Streptomyces griceus* chitosanase was 46.3%; lipase A from *Aspegillus niger* was 42.5%; *Trichoderma viride* cellulase was 46.1%; pork pepsin was 52.2% [69]; *B. cereus* S1 chitosanase was 100% [70]; a combination of chitinase and snailase was 24% [71]; *Aeromonas media* KLU 11.16 chitosanase was 96.14% [72]; and *Bacillus pumilus* BN-262 chitosanase was 40% [73]. This indicated that the COS production yield of *Paenibacillus* sp. TKU047 chitosanase in this study was acceptable. Next, the mixture of COS was analyzed using MALDI-TOF mass spectrometry, a powerful tool for the characterization of COS by giving the information of its degrees of polymerization (DP), as well as the composition units [74]. Chitosan oligomers peaks are shown in Figure 6. The peaks at 363.157 *m/z*, 524.235 *m/z*, and 685.294 *m/z* could be signs for [H(C_6_H_11_O_4_N)_2_OH + Na]^+^, [H(C_6_H_11_O_4_N)_3_OH + Na]^+^, and [H(C_6_H_11_O_4_N)_4_OH + Na]^+^, which indicated for (GlcN)_2_, (GlcN)_3_, and (GlcN)_4_, respectively. The peak at 828.34 *m/z* could be a sign for [H(C_6_H_11_O_4_N)_5_OH − (H_2_O) + Na]^+^ [74], indicating for (GlcN)_5_. The mass differences between 828.34 *m/z* and 989.398 *m/z*; 989.398 *m/z* and 1150.465 *m/z*; 1150.465 *m/z* and 1311.545 *m/z*; and 1311.545 *m/z* and 1473.627 *m/z* were 161 units (approximately), which was a sign for a GlcN unit (C_6_H_11_O_4_N). Thus, the peaks at 989.398 *m/z*, 1150.465 *m/z*, 1311.545 *m/z*, and 1473.627 *m/z* could indicate (GlcN)_6_, (GlcN)_7_, (GlcN)_8_, and (GlcN)_9_, respectively. As such, the MALDI-TOF mass spectrometry result revealed that the COS was (GlcN)_n_ with DP from 2 to 9, in which the major GlcN oligomers were (GlcN)_2_ to (GlcN)_6_. The DDA value and the composition of the obtained COS and 2 commercial COSs (food-grade chitosan oligosaccharide (CCOS_1) and chitosan oligosaccharide X13 (CCOS_2)) from Charming & Beauty Co. (Taipei, Taiwan) were compared, as shown in Table 3. The obtained COS possessed a higher DDA value than commercial COSs (100%, 78.5%, and 74.7%, respectively). Additionally, the composition of the obtained COS differed from commercial COSs. Particularly, the obtained COS contained only homo-COS of GlcN with DP = 2–9, whereas CCOS_1 and CCOS_2 were a mixture of hetero-COS and homo-COS with DP = 2–6. This suggested that the biological activity of those COSs could be dissimilar.

### 3.7. Antioxidant Activity of COS

**Figure 7:**
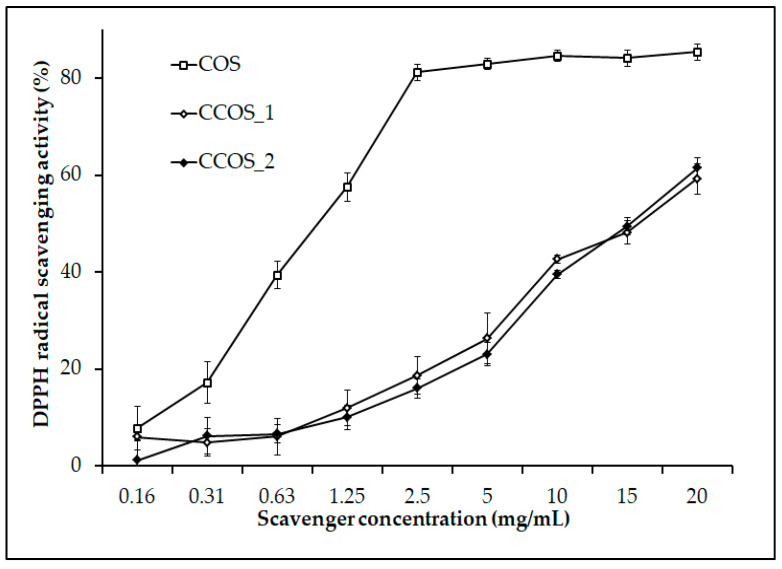
2,2-diphenyl-1-picrylhydrazyl (DPPH) radical scavenging activity of some COSs. All data points were the mean and standard deviation.

Free radicals could cause damage to DNA and protein molecules with their unstable property. Thus, the accumulation of free radicals in the human body could be the cause of many serious diseases, such as neurodegenerative diseases, diabetes, or cancer [75]. A suitable diet that consists of sufficient consumption of antioxidants could defend the body from the adverse impacts of free radicals. In the search of antioxidants for food and medical applications, COS was revealed as a potential candidate by showing good free radical scavenging activity, as well as its natural origin. Furthermore, the antioxidant activity of COS was strongly affected by its molecular weight and degree of deacetylation [76]. Particularly, COS with a lower molecular weight could express higher radical scavenging activity than that with a larger molecular weight. Some research also pointed out that an increase in the DDA value of chitosan could increase the antioxidant activity of this material [76]. As such, it is worth investigating the radical scavenging activity of COS from the chitosan hydrolysis process catalyzed by *Paenibacillus* sp. TKU047 chitosanase, which has a low molecular weight (with DP from 2 to 9) and high DDA (contained only GlcN unit). In this research, the free radical scavenging activity of two commercial COSs (food chitosan oligosaccharide (CCOS_1) and chitosan oligosaccharide X13 (CCOS_2)) from Charming & Beauty Co. (Taipei, Taiwan) was also tested. As shown in Figure 7, all scavengers were observed to be dose-dependent on DPPH radical scavenging activity. Among the COSs, COS produced with *Paenibacillus* sp. TKU047 chitosanase expressed a higher activity than that from the commercial COSs. At 2.5 mg/mL, COS showed 81.20% ± 1.62% of antioxidant activity, whereas CCOS_1 and CCOS_2 were 18.63% ± 3.91%, and 15.96% ± 2.02%, respectively. The DPPH radical scavenging activity IC_50_ value of obtained COS was 1.02 ± 0.05 mg/mL lower, approximately 15-fold lower than that of CCOS_1 (15.37 ± 0.19 mg/mL) and CCOS_2 (15.16 ± 0.39 mg/mL) (Table 4). The lower DPPH radical scavenging activity of commercial COSs may be related to their DDA level. It was found that the free amino group (-NH_2_) on the GlcN unit greatly contributed to the free radical scavenging activity of COS [76]. As a result, there was a great agreement between the result of this study and other reports, which suggested that COS with a higher DDA could give a better DPPH radical scavenging activity [76,77,78]. Consequently, by expressing the greater free radical scavenging activity, COS prepared from the chitosan hydrolysis process catalyzed by *Paenibacillus* sp. TKU047 chitosanase may be considered as an antioxidant candidate in food or medicine fields.

## 4. Conclusions

Chitosanase possessed the potential to be used as an efficiency tool to prepare chitosan oligosaccharide (COS); however, the price of the enzyme was a potential limitation for this application [2,4,60]. Thus, the current study aimed to produce a chitosanase from the reclamation of the squid pens process by *Paenibacillus* sp. TKU047. A chitosanase with 23 kDa of molecular weight, which was supposedly the smallest chitinolytic enzyme from *Paenibacillus* strains, was isolated from the SPP culture medium. Additionally, the COS mixture (DP = 2–9), which was produced from the hydrolysis of 98% DDA chitosan catalyzed by TKU047 chitosanase, revealed the excellent free radical scavenging activity, as compared to other commercial COSs. Results of this study suggested that *Paenibacillus* sp. TKU047 chitosanase could be considered as a suitable enzyme to produce bio-activity COS.

## Figures and Tables

**Table 1:** Purification of *Paenibacillus* sp. TKU047 chitosanase.

Step	Total Protein (mg)	Total Activity (U)	Specific Activity (U/mg)	Recovery (%)	Purification (Fold)
Cultural supernatant	1390.80	111.80	0.08	100.00	1.00
(NH_4_)_2_SO_4_ precipitation	129.64	57.96	0.45	51.84	5.56
FPLC	15.35	29.45	1.92	26.34	23.87
HPLC	0.84	5.45	6.45	4.87	80.28

**Table 2:** Comparison of chitinase/chitosanase produced by *Paenibacillus* strains.

Strain	Stability		Optimum		Molecular Weight	C/N Source	Reference
	pH	Temp.	pH	Temp.			
*Paenibacillus* sp. TKU047	6–9	≤40	7	60	23	SPP	This study
*P. thermoaerophilus* TC22-2b	4–11	≤40	4	60	48	CC ^1^	[38]
*P. dendritiformis*	6–7	20–50	7	45	31		[63]
*P. macerans* TKU029	3–8	≤50	7	60	63	SPP	[13]
*P. mucilaginosus* TKU032	4–8	≤70	6	70	59	SHP	[8]
*Paenibacillus* sp. TKU042					70	SPP	[31]
*Paenibacillus* sp. 1794			4.8		40	Chitosan and COS	[30]
*P. illinoisensis* KJA-424 ^2^			5	60	54	CC	[37]
*P. timonensis* LK-DZ15	2–6		4.5	80	70	CC	[64]
*P. pasadenensis* NCIM 5434			10	37	35	CC	[36]
*P. pasadenensis* CS0611	4–11	≤40	5	50	69	Crab shell and peptone	[39]
*P. barengoltzii* CAU904	3–9	≤55	3.5	60	67		[65]
*P. elgii* HOA73	3–11		7	50	68		[33]
*P. ehimensis* MA2012 ^3^					35, 37, 50, 60, 65, 72, 100, and >100	Crab shell powder, gelatin, complete fertilizer, sucrose, and yeast extract	[59]
*Paenibacillus* sp. D1			5	50	56.56	Urea, chitin, and yeast extract	[34,66]
*P. chitinolyticus* NP-306 ^4^						CC and LB medium	[61]
*Paenibacillus* sp. str. FPU-7 ^5^					61, 78, 82, 87, 97, 122, and 153	Chitin flakes, yeast extract, bonito extract, and peptone	[62]
*Paenibacillus* sp. BISR-047	3–10	35–100	5	55		CC, ammonium sulfate and yeast extract	[32]

^1^ CC: Colloidal chitin. ^2^ Three chitinase activity bands were observed in the culture medium (38, 54, and 63 kD). ^3^ Eight chitinases. ^4^ Thirteen bands of chitinase isozymes on SDS-PAGE. ^5^ Seven chitinases.

**Table 3:** The characteristics of COS, CCOS_1, and CCOS_2.

Name	DDA (%)	Composition
COS	100	(GlcN)_2–9_
CCOS 1	76.5	(GlcN)_2–6_, (GlcNAc)_2_, (GlcNAc)_2_(GlcN)_1–3_, and (GlcNAc)_1_(GlcN)_5_
CCOS_2	74.7	(GlcN)_2–6_, (GlcNAc)_2_, and (GlcNAc)_2_(GlcN)_1–3_

COS: the obtained chitosan oligosaccharide from 98% DDA chitosan hydrolysis process catalyzed by *Paenibacillus* sp. TKU047 chitosanase; CCOS_1: food-grade chitosan oligosaccharide; CCOS_2: chitosan oligosaccharide X13. The DDA value and composition of COSs were analyzed from the MALDI-TOF mass spectrometry results.

**Table 4:** The IC_50_ value of DPPH radical scavenging activity of some COSs.

Scavenger	IC_50_ Value (mg/mL)
COS	1.02 ± 0.05
CCOS_1	15.37 ± 0.19
CCOS_2	15.16 ± 0.39

All data points were the mean and standard deviation.
